# IL-37 protects against airway remodeling by reversing bronchial epithelial–mesenchymal transition via IL-24 signaling pathway in chronic asthma

**DOI:** 10.1186/s12931-022-02167-7

**Published:** 2022-09-13

**Authors:** Kang-ni Feng, Ping Meng, Xiao-ling Zou, Min Zhang, Hai-ke Li, Hai-ling Yang, Hong-tao Li, Tian-tuo Zhang

**Affiliations:** Department of Pulmonary and Critical Care Medicine, The Third Affiliated Hospital of Sun Yat-Sen University, Institute of Respiratory Disease of Sun Yat-Sen University, NO.600 Tianhe Road, Guangzhou, 510630 Guangdong China

**Keywords:** Asthma, Epithelial–mesenchymal transition, Airway remodeling, Interleukin-24, Interleukin -37

## Abstract

**Background:**

Epithelial–mesenchymal transition (EMT) is one of the mechanisms of airway remodeling in chronic asthma. Interleukin (IL)-24 has been implicated in the promotion of tissue fibrosis, and increased IL-24 levels have been observed in the nasal secretions and sputum of asthmatic patients. However, the role of IL-24 in asthmatic airway remodeling, especially in EMT, remains largely unknown. We aimed to explore the effect and mechanism of IL-24 on EMT and to verify whether IL-37 could alleviate IL-24-induced EMT in chronic asthma.

**Methods:**

BEAS-2B cells were exposed to IL-24, and cell migration was assessed by wound healing and Transwell assays. The expression of EMT-related biomarkers (E-cadherin, vimentin, and α-SMA) was evaluated after the cells were stimulated with IL-24 with or without IL-37. A murine asthma model was established by intranasal administration of house dust mite (HDM) extracts for 5 weeks, and the effects of IL-24 and IL-37 on EMT and airway remodeling were investigated by intranasal administration of si-IL-24 and rhIL-37.

**Results:**

We observed that IL-24 significantly enhanced the migration of BEAS-2B cells in vitro. IL-24 promoted the expression of the EMT biomarkers vimentin and α-SMA via the STAT3 and ERK1/2 pathways. In addition, we found that IL-37 partially reversed IL-24-induced EMT in BEAS-2B cells by blocking the ERK1/2 and STAT3 pathways. Similarly, the in vivo results showed that IL-24 was overexpressed in the airway epithelium of an HDM-induced chronic asthma model, and IL-24 silencing or IL-37 treatment could reverse EMT biomarker expression.

**Conclusions:**

Overall, these findings indicated that IL-37 mitigated HDM-induced airway remodeling by inhibiting IL-24-mediated EMT via the ERK1/2 and STAT3 pathways, thereby providing experimental evidence for IL-24 as a novel therapeutic target and IL-37 as a promising agent for treating severe asthma.

**Supplementary Information:**

The online version contains supplementary material available at 10.1186/s12931-022-02167-7.

## Background

Asthma is a heterogeneous chronic disease with the pathological features of variable airflow obstruction, airway hyperresponsiveness, airway inflammation and remodeling [1]. Chronic airway inflammation and repeated tissue repair in response to persistent external environmental stress can induce irreversible airway structural remodeling in asthma [2, 3]. Notably, the features of airway remodeling include epithelial barrier dysfunction, goblet cell metaplasia, airway smooth muscle layer thickening, and angiogenesis, which contribute to steroid-resistant asthma, as well as acute exacerbation of asthma [4–6]. Therefore, identifying effective interventions for the early occurrence and development of airway remodeling is beneficial for improving the prognosis of asthmatic patients.

Epithelial–mesenchymal transition (EMT) is a pathophysiological process in which epithelial cells transform into differentiated mesenchymal cells through phenotypic transformation and is characterized by various changes, including the loss of epithelial cell polarity and the epithelial marker E-cadherin, along with the acquisition of migration capacity and the upregulation of the mesenchymal markers vimentin, α-SMA, N-cadherin and fibronectin [7, 8]. Dysfunctional EMT is considered to be one of the main causes of the mesenchymal cell generation, organ fibrosis, fibrotic tissue repair, and cancer metastasis [9–11]. Emerging evidence has shown that epithelial cell transformation into myofibroblasts is the key mechanism contributing to airway remodeling in severe refractory asthma [5, 12], but the intrinsic factors associated with EMT have not been fully determined.

IL-24, which is also referred to as melanoma-differentiation-associated gene 7 (MDA-7), is a member of the IL-10 cytokine family [13]. As a pleiotropic cytokine, its activity mainly depends on heterodimeric receptor (IL-20RA/IL-20RB and IL-22RA/IL-20RB) expression patterns in target tissues to mediate downstream signaling pathways [14]. Recently, a series of studies have reported the connection between IL-24 and immune inflammatory diseases, including asthma [15], psoriasis [16], systemic lupus erythematosus [17], atopic dermatitis [18], inflammatory bowel disease [19, 20], and rheumatoid arthritis [21]. Despite its extensive antitumor effect [22], several lines of evidence have revealed that IL-24 also shows a profibrotic effect on tissue remodeling. For example, Pap et al. showed that the expression of α‑SMA, fibronectin and TGF-β was lower in the kidneys of IL-20RB KO mice than in those of WT mice [23]. Likewise, decreased expression of fibronectin and collagen I in IL-24^(−/−)^ mice induced by bleomycin was observed compared to that in WT mice [24]. Moreover, a study by Novak et al. showed that all three receptor subunits of IL-24 (IL-20R1, IL-20R2 and IL-22R1) could be detected in human lung epithelial cells [14, 25]. Importantly, IL-24 was overexpressed in the nasal secretions and induced sputum of asthmatic patients [15]. However, little evidence has demonstrated the interaction between IL-24 and EMT-related airway remodeling in asthma, and the potential mechanism remains unclear.

IL-37, which is one of the IL-1 family members, has robust anti-inflammatory effects on innate and adaptive immunity [26]. Our previous work demonstrated that IL-37 alleviated airway eosinophil infiltration and airway remodeling in an HDM-induced murine asthma model [27]. Whether the negative regulator IL-37 could exert a therapeutic effect on IL-24-mediated EMT in the bronchial epithelium, to regulate airway remodeling in asthma remains unclear.

In the current study, to better determine the biological function of IL-24 in asthma, we examined the role and signaling mechanism of IL-24 in BEAS-2B cells, focusing on migration and EMT, and then further elucidated whether IL-37 could inhibit HDM-induced asthmatic airway remodeling by affecting IL-24-induced EMT.

## Methods

### Cell culture

A normal human bronchial epithelial cell line (BEAS-2B) was purchased from American Type Culture Collection (ATCC, USA). The cells were cultured in complete RPMI-1640 medium (Gibco, Thermo Fisher Scientific, USA) supplemented with 10% fetal bovine serum (FBS, Gibco) and 1% streptomycin/penicillin (Gibco) in a humidified incubator containing a 5% CO_2_ atmosphere at 37 °C. The inhibition of JAK/STAT3 and ERK1/2 signaling pathway were referenced previous methods [28, 29]. In short, cells were pretreated with tofacitinib (50 μM, a JAK inhibitor, MCE), PD98059 (20 μM, a ERK1/2 inhibitor, MCE) or DMSO (dilution ratio equal to specific inhibitors) for 1 h, and then exposed to rhIL-24 (100 ng/ml) for 24 h or 48 h. All cell experiments were repeated independently three times.

### Cell viability assay

To determine the cytotoxic effect of IL-24 in BEAS-2B cell**s**, a Cell Counting Kit-8 assay (Beyotime, Shanghai, China) was performed according to the manufacturer’s instructions. Cells were seeded in a 96-well plate (1 × 10^4^/well) and treated with various concentrations of IL-24 (0.1–100 ng/ml, cat# 200-35-20, PeproTech, USA) at 37 °C. After incubation for 24 h, 10 μl CCK-8 solution (Beyotime) was added to each well, and then the cells were incubated for 2 h at 37 °C. Finally, the absorbance at 450 nm was measured using a microplate reader (BioTek, USA). We set five replicate wells per group, and each experiment was repeated three times.

### Cell apoptosis assay

For apoptosis analysis, the cells (5 × 10^5^/well) were seeded in a 6-well plate and treated with 10 or 100 ng/ml IL-24 for 24 h. The single-cell suspensions were collected and incubated with 3 μl Annexin V-FITC and propidium iodide (PI) antibodies (Beyotime) for 15 min at room temperature in the dark for apoptosis analysis using a flow cytometer (BD Biosciences, USA) according to the kit’s procedures. The data represent three independent experiments.

### Cell cycle analysis

For cell cycle analysis, cells (5 × 10^5^/well) were seeded in 6-well plates and treated with 10 or 100 ng/ml IL-24. After 24 h, the cells were harvested and fixed with ice-cold 70% ethanol overnight at 4 °C. The cells were washed twice in ice-cold phosphate-buffered saline (PBS) and subsequently incubated with RNase A/PI solution (Beyotime) for 30 min at room temperature after centrifugation and removal of ethanol. The DNA contents were carried out by flow cytometry. Histograms of DNA were analyzed using ModFit LT software (Verity Software House, USA). The data represent three independent experiments.

### Quantitative real-time-PCR (RT-qPCR)

Total RNA from cultured cells was extracted by RNAiso Plus reagent (Takara, Dalian, China) according to the manufacturer’s protocol. Then, the RNA sample was reverse transcribed into cDNA by PrimeScript RT Master Mix (Takara). RT–qPCR was performed using TB Green Premix Taq (Takara) following the standard procedure on an ABI PRISM 7500 sequence detector (Applied Biosystems, USA). RT–qPCR conditions were as follows: 30 s at 95 °C, followed by 40 cycles of 5 s at 95 °C and 34 s at 60 °C. The relative levels of target genes were compared with the internal reference GAPDH and calculated by the 2^−ΔΔCt^ method. The primers are shown in Table 1. All RT-qPCR experiments were repeated independently three times (n = 3 repeated wells).

**Table 1:** Primer sequences used in this study

Primer	Sequences (5′–3′)	Products (bp)
E-cadherin	(F)5′-CGGGAATGCAGTTGAGGATC-3′ (R)5′-AGGATGGTGTAAGCGATGGC-3′	201
Vimentin	(F)5′-GAGAACTTTGCCGTTGAAGC-3′ (R)5′-GCTTCCTGTAGGTGGCAATC-3′	163
α-SMA	(F)5′-GGTGACGAAGCACAGAGCAA-3′ (R)5′-CAGGGTGGGATGCTCTTCAG-3′	150
GAPDH	(F)5′-GAGTCAACGGATTTGGTCGT-3′ (R)5′-GACAAGCTTCCCGTTCTCAG-3′	185

### Western blot

The proteins of cells or murine right lung tissues were extracted by ice-cold RIPA lysis buffer containing protease and phosphatase inhibitor cocktails (Beyotime) on ice. Protein concentrations were determined with a BCA protein quantification kit (DingGuo, China). The samples (40–70 μg total protein/well) were loaded onto SDS–PAGE gels and then transferred onto PVDF membranes. After blocking with 5% bovine serum albumin (5% BSA, Sigma-Aldrich, USA) for 1 h, the bands were incubated with the appropriate primary antibody (dilution, 1:1000) overnight at 4 °C. Next day, the secondary antibody of goat anti-rabbit or goat anti-mouse IgG linked with HRP (dilution, 1:3000) was incubated for 1 h at room temperature. The bands were visualized with enhanced chemiluminescence (ECL) solution (Merck Millipore, Germany), and the density was quantified using ImageJ software. The relative expression levels were analyzed with β-actin as a loading control. The antibodies were used as follows: anti-E-cadherin (cat# 14472, Cell Signaling Technology, USA), anti-vimentin (cat# 5741, CST), anti-α-SMA (cat# ab32575, Abcam), anti-p-STAT3(cat# 9145, CST), anti-STAT3(cat# 12,640, CST), anti-p-ERK1/2 (cat# 4073, CST), anti-ERK1/2 (cat# 4695, CST), anti-p-p38MAPK (cat# 9215, CST), anti-p38MAPK (cat# 8690, CST), anti-p-NF-κb p65 (cat# 3033, CST), anti-NF-κb p65 (cat# 8242, CST), anti-p-JNK (cat# 4668, CST), anti-JNK(cat# 9252, CST), β-actin (cat# 20536-1-AP, Pepro Tech), anti-rabbit IgG HRP-linked antibody (cat# 7074, CST), and anti-mouse IgG HRP-linked antibody (cat# 7076, CST).

### Wound healing (scratch) assay

Cells were plated on 6-well plates (2 × 10^5^ cells/well) in complete culture medium until 90% confluence. The straight monolayers were scratched with a 10 μl pipette tip on the bottom of the plate and washed three times with PBS. IL-24 (100 ng/ml, PeproTech) with or without IL-37 (100 ng/ml, cat# 200–39-25, PeproTech) was added to the medium and cultured at 37 °C. The area of the scratch was recorded at 0 h, 12 h, and 24 h under an inverted microscope and then quantified using ImageJ software. All wound healing assay was repeated three times. At least three randomly selected fields were calculated, and the average closure area rates were presented.

### Cell migration assay

Cell migration assays were conducted using 24-well Transwell chambers (8.0 μm; Corning, USA). In brief, 600 μl medium (supplemented with 10% FBS) was added to the lower chamber, and 100 μl of serum-free cell suspension (1 × 10^5^ cells) was added to the upper chamber. After IL-24 (100 ng/ml, PeproTech) with or without IL-37 (100 ng/ml, PeproTech) was added to the lower chamber as a chemoattractant, the chambers were incubated at 37 °C for 24 h. After the cell suspension of the upper chamber was removed, the lower chambers were fixed with 4% paraformaldehyde (Biosharp, China) for 1 h and stained with 0.5% crystal violet for 15 min. The migrated cells to the bottom surface were captured using an inverted microscope (Nikon, Japan), and the average number of migratory cells in five randomly selected fields of each well was calculated using ImageJ software. Representative histogram represents three duplicate experiments.

### Immunofluorescence

The treated BEAS-2B cells or murine lung sections were fixed with 4% paraformaldehyde and then permeated with 0.2% Triton X-100 (Solaibio, China) for 30 min. After blocking with 5% BSA for 1 h at room temperature, the cells or slides were incubated with anti-vimentin (1:200, CST), anti-α-SMA (1:200, Abcam), anti-p-STAT3 (1:400, CST), anti-p-ERK1/2 (1:400, CST), anti-IL-1R8(1:50, cat# PA5-20,078, Invitrogen), and anti-IL-18Ra (1:50, cat# MAB840, RD) primary antibodies overnight at 4 °C. The second day, the cells were incubated with Alexa Fluor 488-labeled goat anti-rabbit IgG (1:1000, cat# 4412, CST), Alexa Fluor 488-labeled mouse anti-rabbit IgG (1:1000, cat# 4408, CST), Alexa Fluor 555-labeled goat anti-rabbit IgG (1:1000, cat# 4409, CST) and Alexa Fluor 555-labeled goat anti-mouse IgG (1:1000, cat# 4413, CST) for 1 h in the dark and subsequently counterstained with DAPI (Beyotime) for nuclear staining. Images were captured under a fluorescence microscope (Nikon, Japan). Five random fields of each slide were selected for quantification of fluorescence intensity.

### Animal experiments

Wild-type (WT) SPF BALB/c mice (female, 6–8 weeks, 20–25 g) were purchased from Yancheng Biotechnology Co., Ltd [Guangzhou, China, license number: SCXK (liao) 2020-0001], and maintained under SPF conditions with regular food and water supplementation under a 12 light/dark cycle at 22 ± 2 °C. Animal experimental procedures were approved by the Ethics Committee of Animal Experiments of the Third Affiliated Hospital of Sun Yat-sen University.

### Antigen challenged protocol

Mice were randomly assigned to five groups (*n* = 8/group): (1) PBS group (control group); (2) HDM group (asthma group); (3) HDM plus si-IL-24 group (asthma + si-IL-24 group); (4) HDM plus si-NC group (asthma + si-negative control group); and (5) HDM plus IL-37 group (asthma + IL-37 group). To establish a chronic allergic asthma model, the mice were delivered intranasally with 25 μg of house dust mite (HDM) extract (cat. No. XPB82D3A2.5, Geer) dissolved in 10 μl PBS, 5 days per week for five consecutive weeks, as previously described [27]. From the third week, mice in group 3 or group 4 were treated with si-IL-24 or si-NC (1 nmol/mouse/day, RiboBio Biotechnology, Guangzhou, China), respectively, intranasally 1 h prior to HDM challenge, three times per week for 3 weeks. The mice in group 5 received rhIL-37 intranasally (1 μg/mouse/day, Pepro Tech) 1 h prior to HDM challenge, three times per week for 3 weeks. Apart from that, the control group mice were administered an equal amount of PBS at the same time as the negative control. All mice were euthanized 24 h after the last exposure. The small interfering (si)RNA sequence for silencing IL-24 in vivo: 5′-GACCUGGAUGCAGAAAUUCUAtt-3′, 5′-UAGAAUUUCUGCAUCCAGGUCtt-3′.

### Assessment of airway hyperresponsiveness (AHR)

Airway resistance of mice was measured using a Buxco® FinePointe™ RC system (DSI, USA) within 24 h after the last HDM challenge. Mice were anesthetized with 1% pentobarbital sodium (60 mg/kg, intraperitoneal injection) and intubated intratracheally to connect to an animal ventilator (120 breaths/min) following exposure to aerosolized saline or increasing doses of methacholine (6.25–50 mg/ml, Sigma–Aldrich). Mice were nebulized for 180 s at each dose and for 30 s intervals in whole-body plethysmography. Then, the average Penh values reflecting airway resistance were detected and analyzed from each group (8 mice per group).

### Analysis of bronchoalveolar lavage fluid (BALF)

The tracheas were flushed with 0.5 ml precooled PBS three times after tracheal cannula. The supernatant of BALF was harvested and centrifuged at 3000 r/min for 5 min at 4 °C, followed by storage at -80 °C for further ELISA analysis. After lysis of RBCs, the total number of cells was counted with a hemocytometer and then the remaining suspension was prepared onto slide preparations by a centrifugal machine for further Diff-Quick staining (TBD, Tianjin, China). At least 400 cells/slide were counted to analyze the percentage of macrophages, eosinophils, neutrophils and lymphocytes under a light microscope at × 200 magnification. For ELISA, the level of active TGF-β1 in BALF was measured by commercial ELISA kits (cat#70-EK981-96, Multisciences Biotech, China) according to the manufacturer’s protocol.

### Histopathological analysis

Mouse lung sections (5 μm) were deparaffinized and rehydrated, and then hematoxylin and eosin (H&E) staining, periodic acid-Schiff (PAS) staining and Masson’s trichrome (Masson) staining were performed to assess the extent of inflammation, mucus production and collagen deposition in murine lung tissues according to the manufacturer’s instructions. The images were captured with a light microscope. The airway inflammation score was quantified according to previously published methods [30]. The quantification of PAS staining and Masson staining were performed by ImageJ software.

### Immunohistochemistry (IHC)

IHC was performed on paraffin-embedded sections (5 μm) from murine left lung tissues. After deparaffinization and rehydration, the slides were incubated in 3% H_2_O_2_ for inactivation of endogenous peroxide and permeabilized with 1% Triton-X (Solarbio, China). The slides were preincubated in citrate buffer and heated for 15 min in a pressure cooker for antigen retrieval. After blocking with 5% BSA, the tissue sections were incubated with anti-rabbit IL-24 antibody (1:100, Pepro Tech) and anti-mouse E-cadherin antibody (1:400, CST) at 4 °C overnight. Next, the slides were incubated with the appropriate HRP-labeled goat anti-rabbit/anti-mouse IgG for 1 h. Brown staining was visualized using diaminobenzidine (DAB) solution (Solarbio). Finally, the slides were counterstained with hematoxylin and dehydrated by xylene as well as a grade alcohol series. The slides were sealed and visualized by a light microscope. The average positive brown area of each slice was analyzed through five randomly selected HPF fields using ImageJ software.

### Statistical analysis

Data are presented as the mean ± standard deviation (SD) and were analyzed in triplicate or more. Statistical analysis was performed by GraghPad Prism 9.0. Student’s t test and one-way ANOVA were used for significant differences between two or multiple groups, respectively. LSD-*t* test was used for comparison between groups. In all cases, *P* < 0.05 was considered statistically significant.

## Results

### The effect of IL-24 on the proliferation, apoptosis and cell cycle of BEAS-2B cells

To investigate the cytotoxicity of IL-24 in bronchial epithelial cells, different concentrations of IL-24 were added to BEAS-2B cells for 24 h and then analyzed by CCK-8 and Annexin V/PI assays. No significant differences were observed regarding proliferation (Additional file 1: Fig. S1a) and apoptosis (Additional file 1: Fig. S1b–d) between the IL-24-treated group and the control group. To further confirm the effect of IL-24 on the cell cycle status, the cells were incubated with different concentrations of IL-24 (10 and 100 ng/ml) for 24 h. As shown in Additional file 1: Fig. S1e and f, exposure to IL-24 had no significant effect on the cell cycle DNA content. The above results suggested that IL-24 has no significant effect on the proliferation and apoptosis of normal bronchial epithelial cells. Combined with the results of the preliminary experiment, 100 ng/ml IL-24 was selected as the final concentration for subsequent in vitro cell experiments.

### IL-24 promoted EMT in BEAS-2B cells

**Fig. 1:**
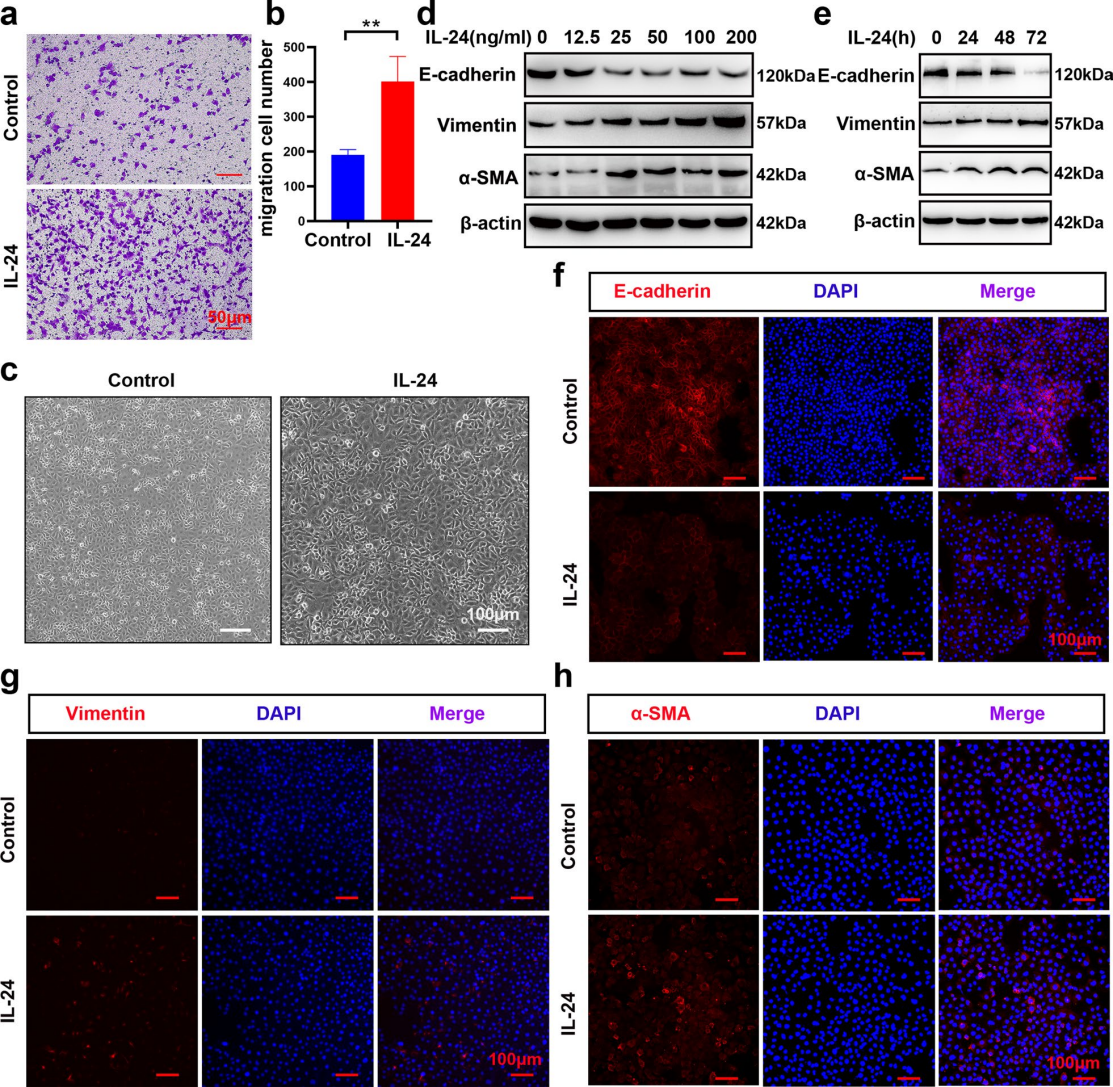
IL-24 promoted cell migration and EMT in BEAS-2B cells. **a** The effect of IL-24 (100 ng/ml) on the migration of BEAS-2B cells was evaluated by Transwell assay. Scale bar = 50 μm (× 100). **b** Quantification of the Transwell assay revealed the number of migrated cells. **c** Representative images of cell morphology following 100 ng/ml IL-24 stimulation for 48 h. Scale bar = 100 μm (× 200). **d** Representative immunoblot analysis of E-cadherin, vimentin and α-SMA expression in BEAS-2B cells treated with various concentrations of IL-24 (0–200 ng/ml) for 48 h. **e** Representative immunoblot analysis of E-cadherin, vimentin, and α-SMA expression in BEAS-2B cells treated with 100 ng/ml IL-24 at different time points. Representative immunofluorescence of E-cadherin (**f**), vimentin (**g**) and α-SMA (**h**) expression in BEAS-2B cells exposed to 100 ng/ml IL-24 for 48 h. Scale bar = 100 μm (× 200). Bar diagrams and data are presented as the mean ± standard deviation (SD) from three replicate experiments. *vs. control group; **P < 0.01

Accumulating evidence supports that the EMT process of bronchial epithelium is closely related to airway remodeling [8, 31, 32]. To further investigate the action of IL-24 on the migration capacity of BEAS-2B cells, Transwell assays and wound healing assays were performed. As illustrated in Fig. 1a and b, a dramatic enhancement in the migratory capability of BEAS-2B cells was observed in the IL-24-treated group compared with the control group. This result was further verified by wound healing assays (Additional file 1: Fig. S2a and b). The typical changes in the EMT process include epithelial cells losing epithelial markers and polarity while acquiring mesenchymal morphology with an increase in mesenchymal markers. To further evaluate the role of IL-24 during the EMT process, cells were incubated with 100 ng/ml IL-24 for up to 48 h to observe morphological changes. As shown in Fig. 1c, the IL-24-treated group showed an acquisition of a larger and more spindle-shaped morphology. Next, we estimated the effect of IL-24 on the expression of EMT-related genes in BEAS-2B cells at different concentrations and time points. Western blot results indicated that IL-24 significantly enhanced mesenchymal marker (vimentin and α-SMA) production, accompanied by a decrease in E-cadherin levels in a dose-dependent (Fig. 1d) and time-dependent manner (Fig. 1e). Likewise, the contribution of IL-24 stimulation to EMT marker (E-cadherin, vimentin, and α-SMA) expression was confirmed by immunofluorescence staining (Fig. 1f-h). Hence, these results indicated that IL-24 could promote the migration ability and EMT process in BEAS-2B cells, which might contribute to the dysregulation of EMT in asthma.

### ERK1/2 and STAT3 signaling pathways were essential for IL-24-induced EMT in BEAS-2B cells

**Fig. 2:**
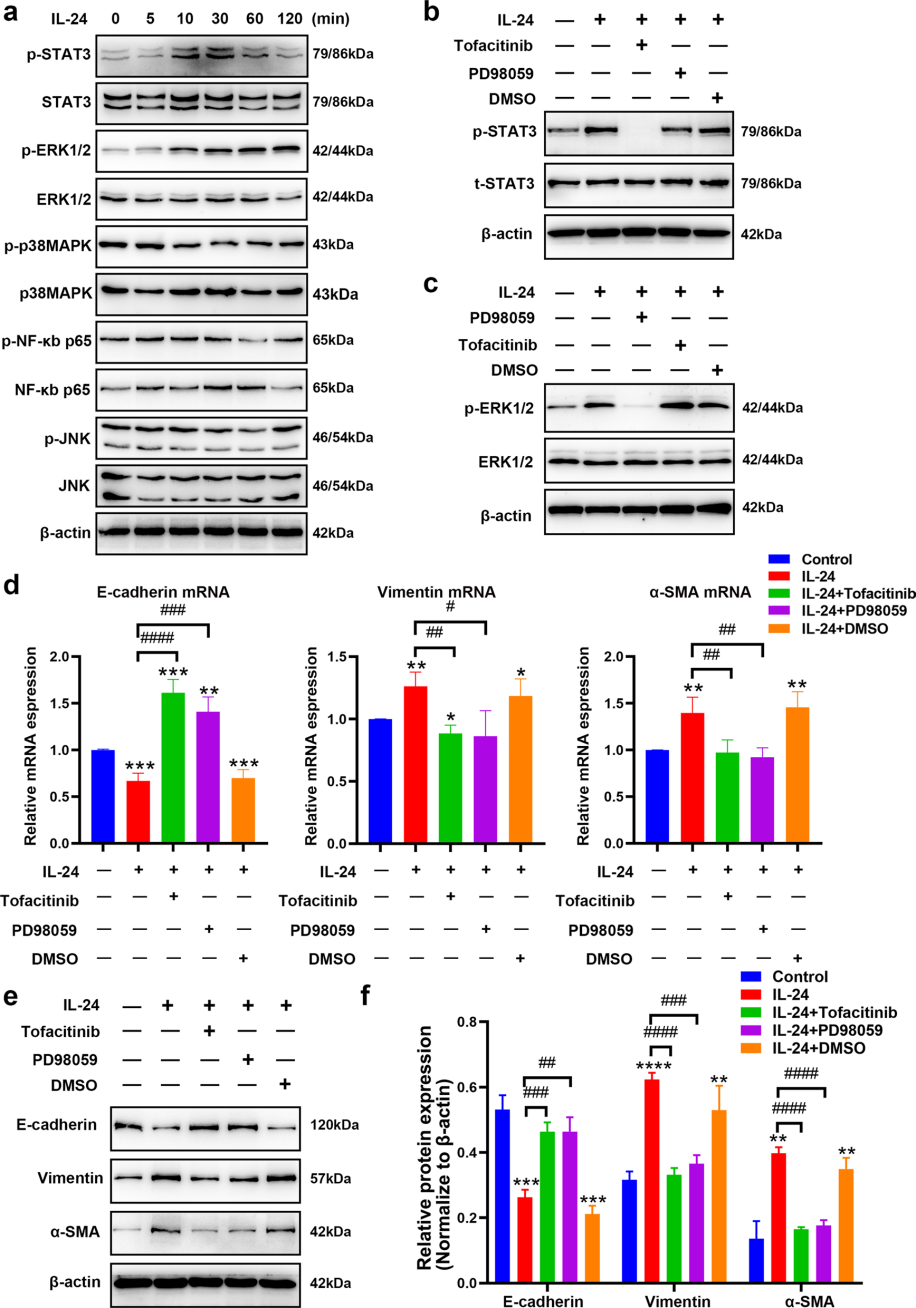
IL-24 induced EMT in BEAS-2B cells via the ERK1/2 and STAT3 pathways. **a** BEAS-2B cells were treated with 100 ng/ml IL-24 at different time points, and the activation of STAT3, ERK1/2, p38MAPK, NF-κb p65 and JNK signaling pathways was analyzed by western blot. **b** The activation of the p-STAT3/STAT3 pathway was analyzed by western blot. BEAS-2B cells were treated with 100 ng/ml IL-24 for 48 h with or without tofacitinib, PD98059 or DMSO pretreatment for 1 h. **c** The activation of the p-ERK1/2/ERK1/2 pathway was analyzed by western blot. BEAS-2B cells were treated with 100 ng/ml IL-24 for 48 h with or without tofacitinib, PD98059 or DMSO pretreatment for 1 h. **d** Quantification of E-cadherin, vimentin and α-SMA mRNA expression in BEAS-2B cells treated with 100 ng/ml IL-24 for 24 h with or without tofacitinib, PD98059 or DMSO pretreatment for 1 h by RT–qPCR. Representative immunoblot analysis (**e**) and quantification (**f**) of E-cadherin, vimentin and α-SMA protein expression in BEAS-2B cells exposed to 100 ng/ml IL-24 for 48 h with or without tofacitinib, PD98059 or DMSO pretreatment for 1 h. Bar diagrams and data are presented as the mean ± standard deviation (SD) from three replicate experiments. Beas-2B cells were treated with completed 1640 culture medium alone as a control group. *vs. control group; ^#^ vs. IL-24 group. *^,#^*P* < 0.05; **^,##^*P* < 0.01; ***^,###^*P* < 0.001; ****^,####^*P* < 0.0001

Previous studies have reported that IL-24 activates multiple pathways, including JAK/STAT3, ERK1/2 and other pathways [20, 33, 34]. We first evaluated their total and phosphorylation levels in BEAS-2B cells treated with IL-24 at different time points by western blot. Here, the results revealed that IL-24 selectively induced STAT3 and ERK1/2 phosphorylation in a time-dependent manner in BEAS-2B cells (Fig. 2a). To further address whether IL-24 instigated EMT by activating the ERK1/2 or STAT3 signaling pathway, we treated BEAS-2B cells with the specific molecular inhibitors tofacitinib (a specific inhibitor of JAK, an upstream protein of STAT3) or PD98059 (a specific inhibitor of ERK1/2,) for 1 h prior to IL-24 stimulation to affect the activation of pathways. As expected, the activation levels of p-STAT3 and p-ERK1/2 signaling pathway induced by IL-24 were effectively inhibited by tofacitinib (Fig. 2b) and PD98059 (Fig. 2c), respectively. Furthermore, tofacitinib or PD98059 pretreatment reduced the level of vimentin and α-SMA mRNA upregulated by IL-24 and restored the loss of E-cadherin mRNA levels (Fig. 2d). A similar trend was also substantiated by western blot analysis; the effect of IL-24 on the upregulation of EMT markers was partly blocked by PD98059, and a relatively weaker analogous trend was observed when tofacitinib was administered (Fig. 2e and f). In general, these data verified that IL-24 acted on the regulation of EMT via STAT3- and ERK1/2-dependent mechanisms in BEAS-2B cells.

### IL-37 inhibited IL-24-induced EMT via the STAT3 and ERK1/2 pathways in BEAS-2B cells

**Fig. 3:**
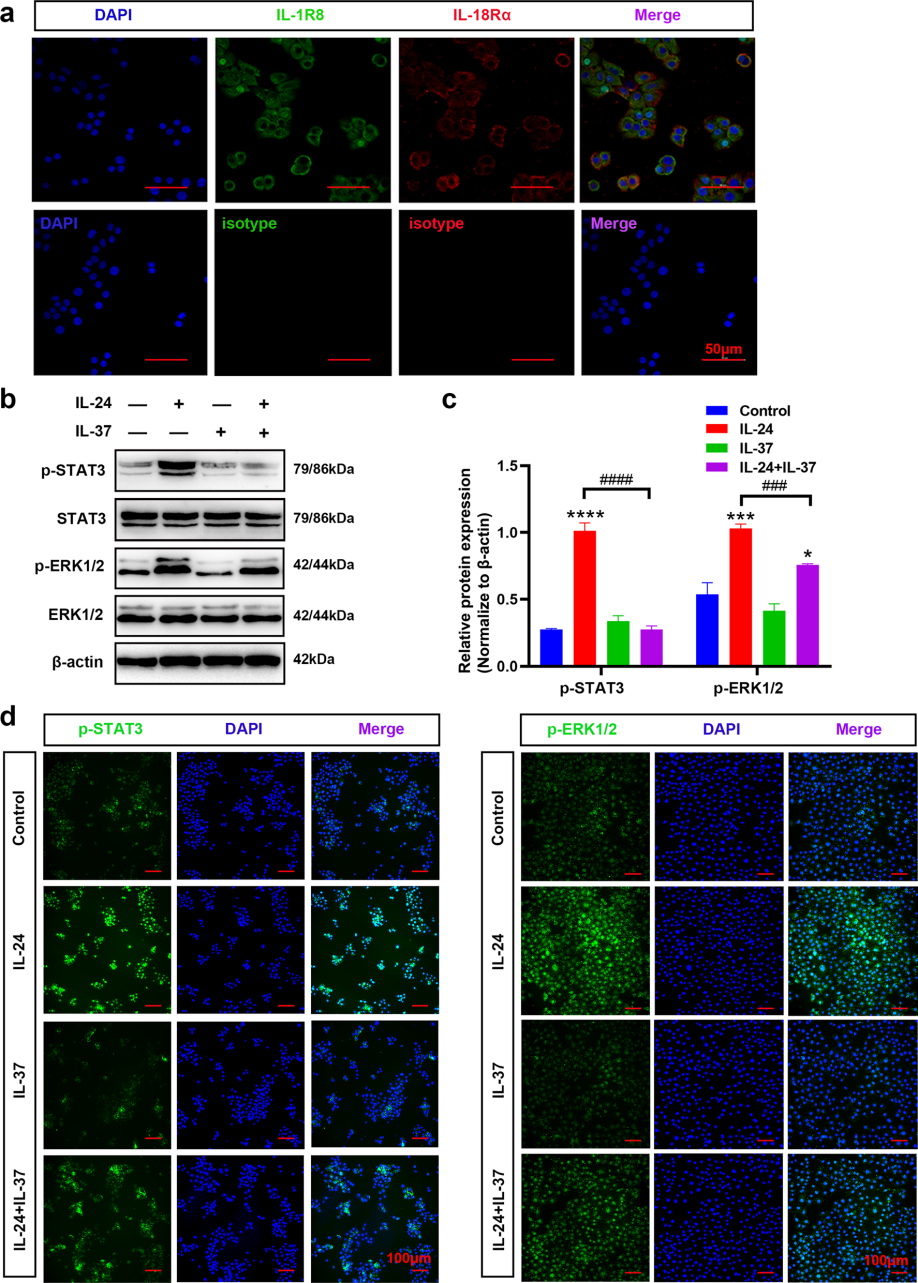
IL-37 inhibited IL-24-mediated EMT by blocking STAT3 and ERK1/2 pathway activation. **a** Representative immunofluorescence of IL-37 receptors (IL-1R8 and IL-18Rα) in BEAS-2B cells. DAPI, blue; IL-1R8, green; and IL-18Rα, red. Scale bar = 50 μm (× 400). Representative immunoblot analysis (**b**) and quantification (**c**) of STAT3 and ERK1/2 signaling pathways in BEAS-2B cells treated with 100 ng/ml IL-24 for 48 h with or without 100 ng/ml IL-37 pretreatment for 8 h. **d** Representative immunofluorescence of p-STAT3 and p-ERK1/2 in BEAS-2B cells treated with 100 ng/ml IL-24 for 24 h with or without 100 ng/ml IL-37 pretreatment for 8 h. DAPI, blue; p-STAT3 and p-ERK1/2, green. Scale bar = 100 μm (× 200). Bar diagrams and data are presented as the mean ± standard deviation (SD) from three replicate experiments. * vs. control group; ^#^ vs. IL-24 group. *^,#^*P* < 0.05; **^, ##^*P* < 0.01; ***^,###^*P* < 0.001, ****^,####^*P* < 0.0001

To further explore whether the inhibitor IL-37 modulates the IL-24-induced activation of the STAT3 and ERK1/2 signaling pathways. We first observed that the surface of BEAS-2B cells constitutively expressed IL-37 receptors (IL-1R8 and IL-18Rα), which enabled the cells to respond to IL-37 (Fig. 3a). As shown in Fig. 3b and c, pretreatment with IL-37 prior to IL-24 stimulation remarkably diminished the elevated phosphorylation levels of STAT3 and ERK1/2 compared to IL-24 exposure alone. Consistently, immunofluorescence staining confirmed that IL-24 promoted p-STAT3 and p-ERK1/2 translocation into the nucleus, and the immunofluorescence intensity of p-STAT3 and p-ERK1/2 induced by IL-24 was significantly attenuated upon exogenous IL-37 treatment (Fig. 3d). Accordingly, these findings demonstrated that IL-24 selectively induced the activation of STAT3 and ERK1/2 signaling pathways in BEAS-2B cells, which could be partly blocked by IL-37 administration.

**Fig. 4:**
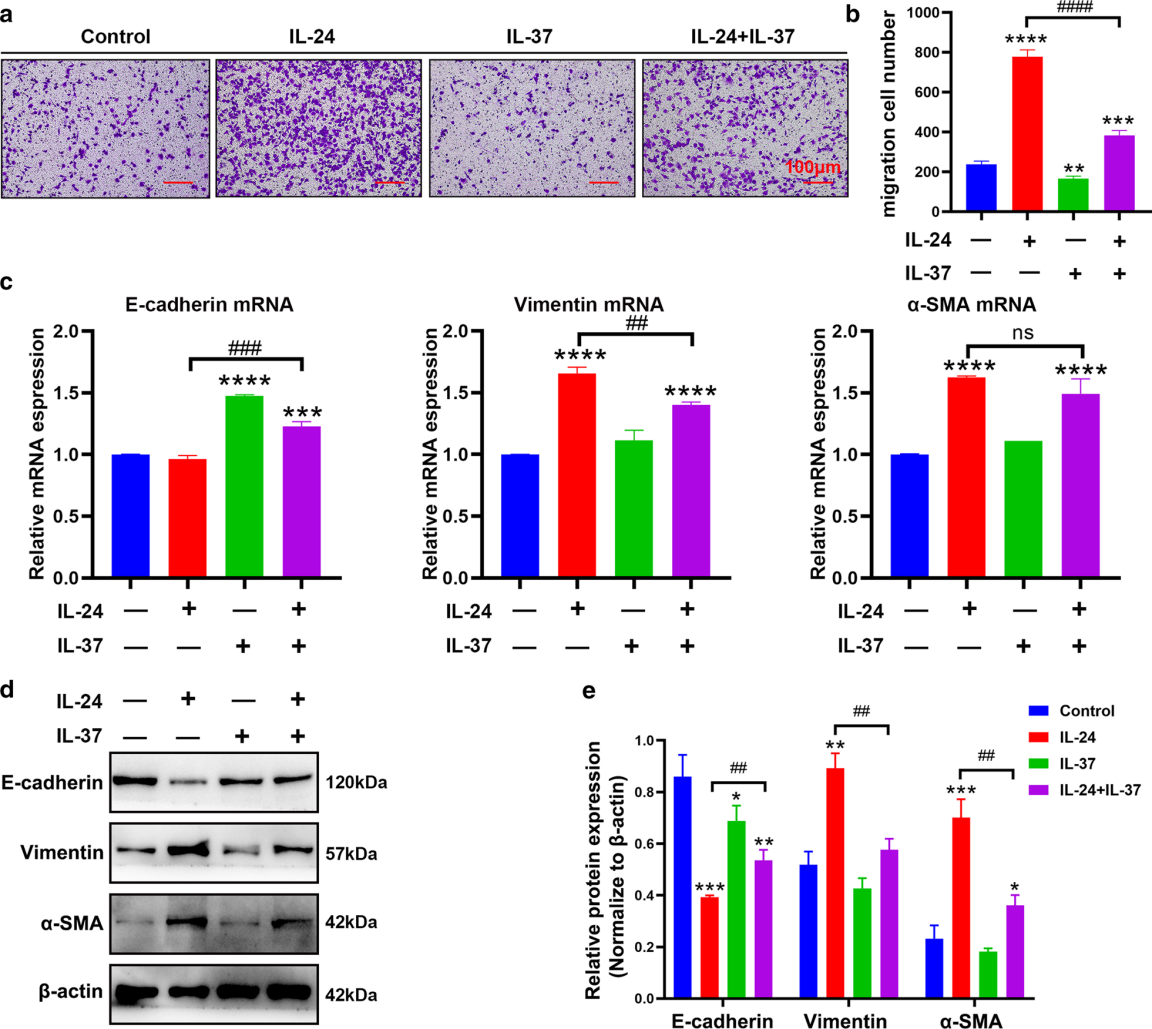
IL-37 attenuated IL-24-induced bronchial epithelial migration and EMT in vitro. **a** The effect of IL-24 with or without IL-37 on the migration of BEAS-2B cells was evaluated by Transwell assay. Scale bar = 100 μm (× 100). **b** Quantification of the Transwell assay showed the number of migrated cells. **c** Quantification of E-cadherin, vimentin and α-SMA mRNA expression in BEAS-2B cells treated with 100 ng/ml IL-24 for 24 h with or without 100 ng/ml IL-37 pretreatment for 8 h by RT–qPCR. Representative immunoblots (**d**) and quantification (**e**) of E-cadherin, vimentin, and α-SMA protein expression in BEAS-2B cells treated with 100 ng/ml IL-24 for 48 h with or without 100 ng/ml IL-37. Bar diagrams and data are presented as the mean ± standard deviation (SD) from three replicate experiments. * vs. control group; ^#^ vs. IL-24 group. *^,#^*P* < 0.05; **^, ##^*P* < 0.01; ***^,###^*P* < 0.001, ****^,####^*P* < 0.0001

In the light of IL-37 played a negative role in IL-24-induced p-STAT3 and p-ERK1/2 phosphorylation levels, and IL-24-induced EMT was dependent on STAT3 and ERK1/2 signaling pathways, we hypothesized that IL-37 may exert an inhibitory effect during the IL-24-induced EMT process and migration in BEAS-2B cells. To address these issues, we initially performed wound healing assays and Transwell assays to evaluate the effect of IL-37 on IL-24-induced migration in BEAS-2B cells. As shown in Fig. 4a and b, the migrated cells were significantly eliminated in the IL-24 + IL-37 cotreated group compared with the IL-24-treated alone group. Similar results were observed in wound healing experiments (Additional file 1: Fig. S2c and S2d). As indicated by RT–qPCR, IL-37 reversed the expression of vimentin and α-SMA induced by IL-24, while preventing the downregulation of E-cadherin (Fig. 4c). Likewise, the western blot results showed a similar trend (Fig. 4d and e). Collectively, these findings supported the speculation that IL-37 could restrain the IL-24-triggered EMT process by inhibiting the p-STAT3 and p-ERK1/2 signaling pathways in BEAS-2B cells.

### Silencing IL-24 or IL-37 treatment mitigated airway inflammation, AHR and airway remodeling in an HDM-induced asthma murine model

**Fig. 5:**
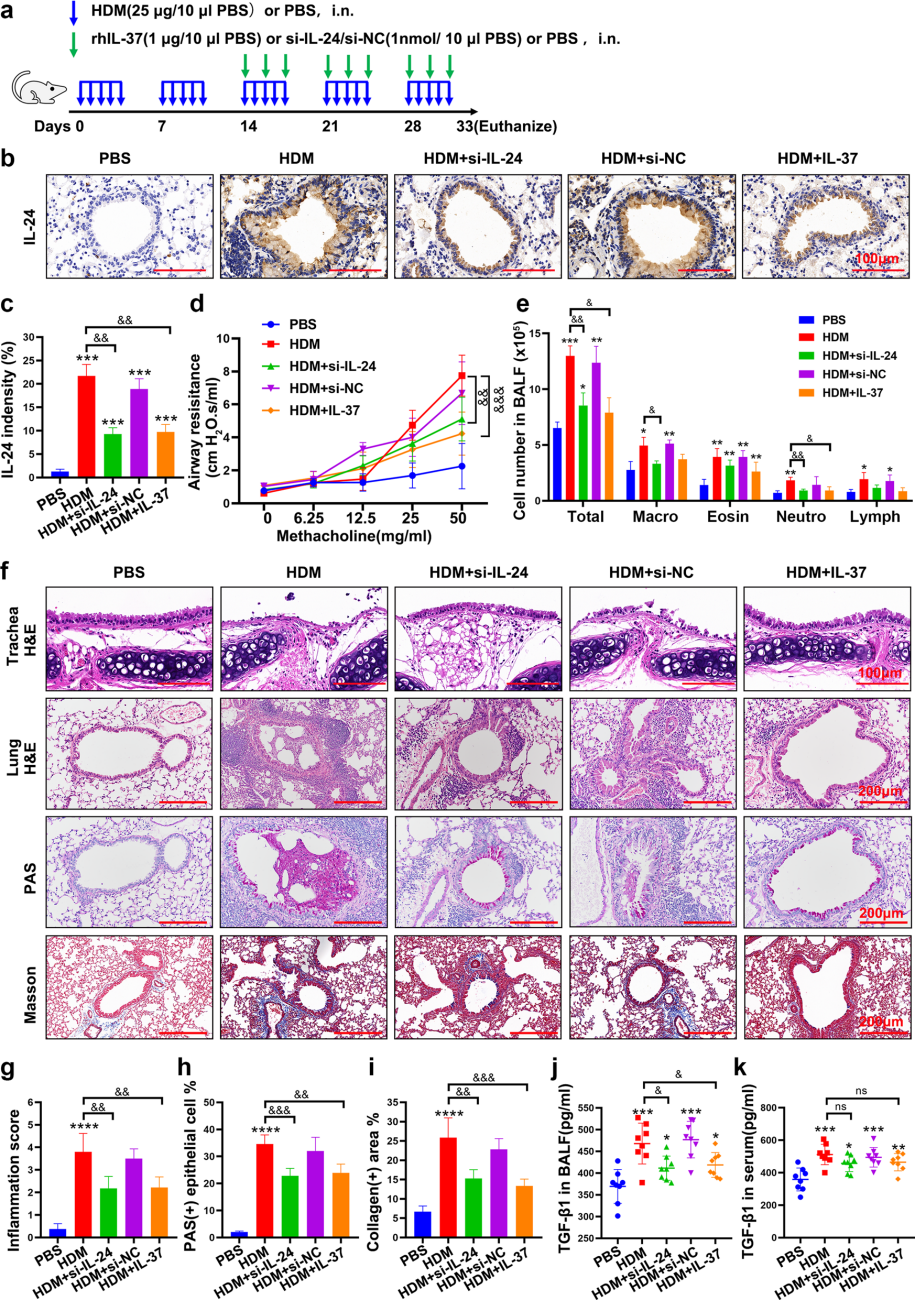
Silencing of IL-24 or IL-37 alleviated AHR, airway inflammation and airway remodeling in an HDM-induced asthma murine model. **a** Schematic diagram of the animal experimental protocol for the chronic asthma murine model (n = 8 mice per group). Representative immunohistochemical images (**b**) and quantification (**c**) of IL-24 in murine lung tissues. Scale bar = 100 μm (× 630). **d** Airway resistance responses to various doses of inhaled methacholine (0–50 mg/ml) were determined within 24 h after the final HDM challenge. **e** The total and differential inflammatory cell counts in the bronchoalveolar lavage fluid (BALF) from each group were determined by Diff-Quick staining. Macro, macrophage; Eosin, eosinophil; Neutro, neutrophil; Lymgh, lymphocyte. **f** Representative histopathological analysis of trachea and lung tissues from mice was performed with H&E staining, PAS staining and Masson staining. H&E staining of trachea sections: scale bar = 100 μm (× 630). H&E, PAS and Masson staining of lung sections: scale bar = 200 μm (× 200). (n = 8/group). Quantification of H&E staining (**g**), PAS staining (**h**) and Masson staining (**i**) in each group. The concentration of active TGF-β1 in the BALF (**j**) and serum (**k**) from each group of mice (n = 8/group). Bar diagrams and data are presented as the mean ± standard deviation (SD). ns, no significant differences. * vs. PBS group; & vs. HDM group. *^,&^*P* < 0.05; **^,&&^*P* < 0.01, ***^,&&&^*P* < 0.001, ****^,&&&&^*P* < 0.0001. si-NC: siRNA targeting of negative control

In this study, we developed an asthma animal model according to a previous method [27], and the protocol is shown in Fig. 5a. To better understand the role of IL-24 in vivo, we first detected the expression of IL-24 in lung sections. The results showed that IL-24 was mainly expressed in bronchial epithelium. After intranasal si-IL-24 administration, the relative IL-24 expression was effectively inhibited in the HDM-sensitized murine asthma model (Fig. 5b and c). Next, lung function results showed that both si-IL-24 and IL-37 markedly attenuated airway resistance at Mch doses of 25 and 50 mg/ml compared with the asthma model group (Fig. 5d). Moreover, downregulation of IL-24 or IL-37 administration induced a decline in the population of total cells, macrophages, eosinophils and neutrophils in the BALF in HDM-sensitized mice (Fig. 5e). In addition, pathological staining results indicated that HDM-induced inflammatory cell infiltration, goblet cell hyperplasia, and collagen fiber deposition in the peribronchial and perivascular areas were remarkably reduced in the si-IL-24 or IL-37 treatment group (Fig. 5f–i).

Since it is generally accepted that TGF-β1 is a pivotal driver of asthma remodeling pathogenesis, we also detected the level of active TGF-β1 in murine serum and BALF. Si-IL-24 or IL-37 treatment appeared to decrease HDM-induced active TGF-β1 levels in the BALF but not in the serum (Fig. 5j and k). These data suggested that intranasal administration of si-IL-24 or IL-37 in local airway tissues might ameliorate the severity of asthma in the lungs.

### Silencing IL-24 or IL-37 treatment decreased the activation of the STAT3 and ERK1/2 signaling pathways in the lungs in an HDM-induced asthma murine model

**Fig. 6:**
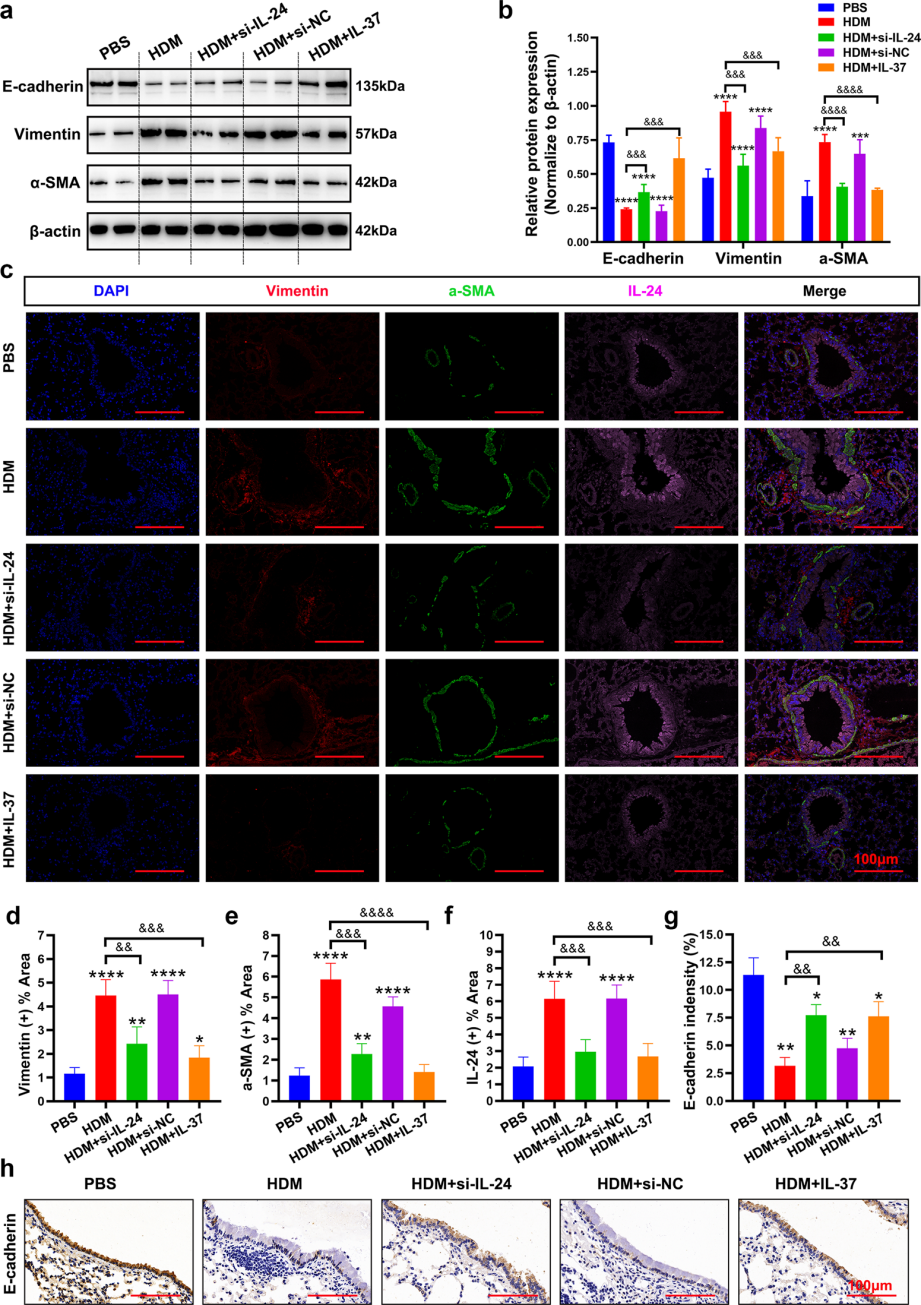
Intranasal administration of si-IL-24 or IL-37 reduced HDM-induced EMT markers in an HDM-sensitized asthma murine model. Representative immunoblot analysis (**a**) and quantification (**b**) of E-cadherin, vimentin and α-SMA protein expression in the lungs of mice. **c–f** Representative immunofluorescence images of α-SMA, vimentin and IL-24 expression in the lungs of mice. Scale bar = 100 μm (× 400). **g** and **h** Representative immunohistochemistry images of E-cadherin expression in the lungs of mice (n = 5 random fields per group). Scale bar = 100 μm (× 630). Bar diagrams and data are presented as the mean ± standard deviation (SD). * vs. PBS group; & vs. HDM group. *^,&^*P* < 0.05; **^,&&^*P* < 0.01; ***^,&&&^*P* < 0.001, ****^,&&&&^*P* < 0.0001. *si-NC* siRNA targeting of negative control

Considering the crucial role of EMT in the pathogenesis of airway remodeling, we analyzed the expression of EMT markers in the lung tissues of mice. Western blot results showed that silencing IL-24 or IL-37 treatment attenuated the production of vimentin and α-SMA, but markedly prevented the decrease in E-cadherin expression after induction of HDM, as shown in Fig. 6a and b. Similar trends can be observed in immunohistochemistry and immunofluorescence analysis. HDM-induced asthmatic mice exhibited a significant upregulation of vimentin and α-SMA expression accompanied by a loss of E-cadherin compared with the negative control group, and these changes could be partly reversed in the si-IL-24 or IL-37 group (Fig. 6c–h).

**Fig. 7:**
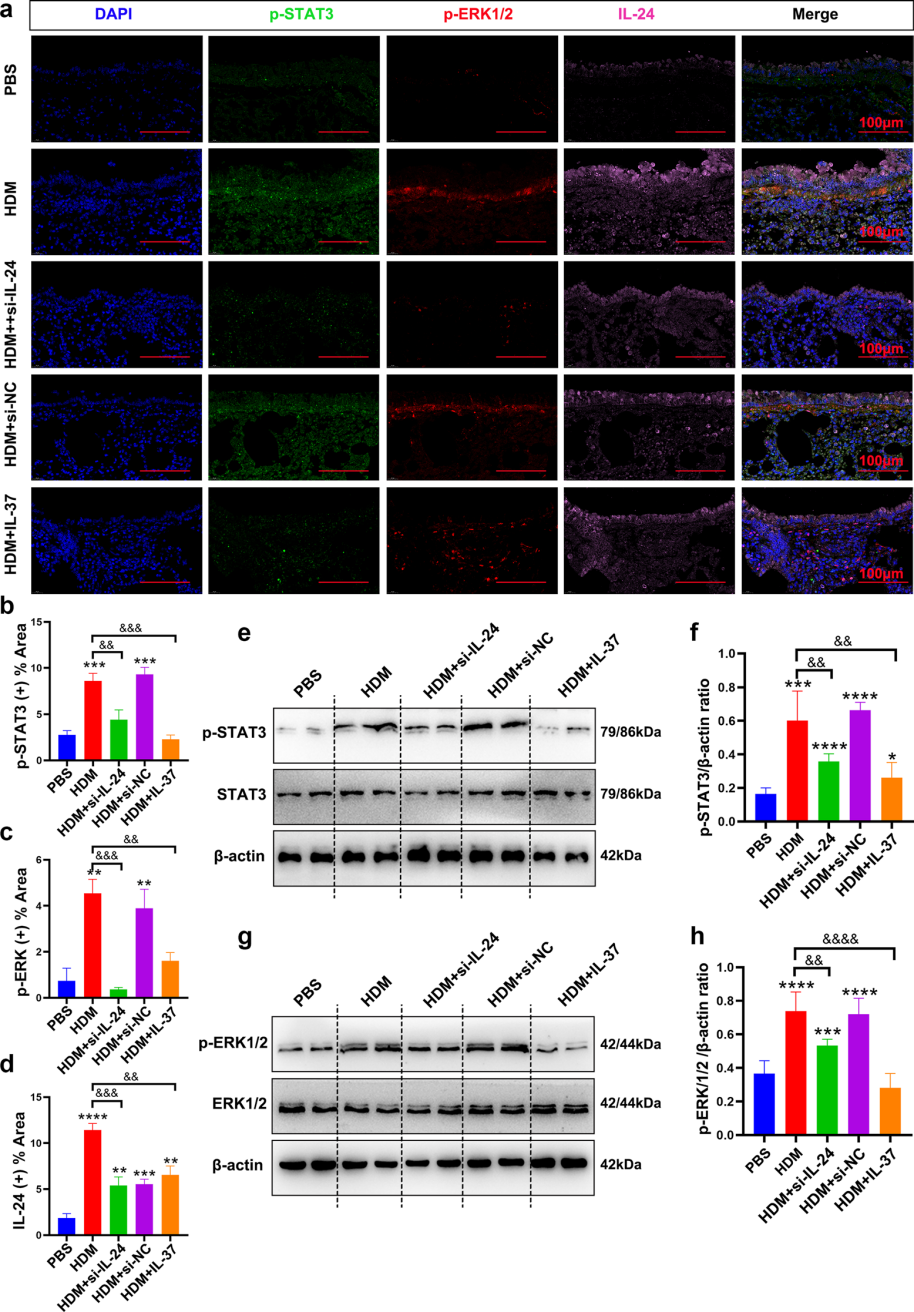
Intranasal administration of si-IL-24 or IL-37 inhibited the activation of the p-STAT3 and p-ERK1/2 signaling pathways in the lungs of an HDM-induced asthma murine model. **a**–**d** Representative immunofluorescence images of p-STAT3 and p-ERK1/2 expression in mouse lung tissues. Scale bar = 100 μm (× 630). **e**–**h** Representative immunoblot analysis and quantification of p-STAT3 and p-ERK1/2 expression in lung tissues of mice (n = 8/group). Bar diagrams and data are presented as the mean ± standard deviation (SD) from three replicate experiments. * vs. PBS group; ^&^ vs. HDM group. *^,&^*P* < 0.05; **^,&&^*P* < 0.01; ***^,&&&^*P* < 0.001, ****^,&&&&^*P* < 0.0001. *si-NC* siRNA targeting of negative control

To further confirm whether IL-37 attenuates HDM-induced EMT through STAT3 and ERK1/2 signaling pathways in vivo, we analyzed the activation of STAT3 and ERK1/2 pathways by immunofluorescence and western blot assays. The results showed that in mice treated with HDM alone, the levels of p-STAT3 and p-ERK1/2 were mainly overexpressed in the airway epithelium and subepithelial layer compared with those in the PBS group (Fig. 7a). Meanwhile, we found that the activation of p-STAT3 and p-ERK1/2 in the epithelium was effectively inhibited in the si-IL-24-treated group or IL-37-treated group (Fig. 7a–d). Western blot data further verified this result (Fig. 7e–h). Overall, these results suggested that the inhibitory effect of IL-37 on the occurrence of EMT and remodeling in asthma may be related to its regulation of IL-24 levels by influencing the activation of the p-STAT3 and p-ERK1/2 signaling pathways.

## Discussion

As an important characteristic of asthma, airway remodeling is one of the important mechanisms that causes AHR and airflow obstruction and is an important reason for the insensitivity of chronic asthma patients to ICS therapy. In the present study, we demonstrated that IL-24 induced cell migration and the mesenchymal phenotype via the STAT3 and ERK1/2 pathways in BEAS-2B cells, and that inhibiting ERK1/2 or STAT3 pathway phosphorylation significantly impaired IL-24-mediated EMT-related protein expression. In addition, we verified the inhibitory effect of IL-37 on BEAS-2B cell migration and EMT in response to IL-24 by blocking the activation of the ERK1/2 and STAT3 pathways. In vivo, we found that IL-24 was highly expressed in the mouse airway epithelium in an HDM-induced asthma model, which was accompanied by the upregulation of EMT markers. Intranasal silencing of IL-24 or IL-37 prevented HDM-induced asthmatic airway inflammation and remodeling by inhibiting EMT marker expression. Our results provide the first evidence that IL-24 contributes to asthmatic airway remodeling by promoting EMT in bronchial epithelial cells and indicate that IL-37 may improve airway remodeling by abrogating IL-24-mediated EMT by inhibiting p-STAT3 and p-ERK1/2 signaling pathway activation, thereby providing new targets for asthma treatment focused on EMT-related airway remodeling.

Our data revealed that IL-24 had no significant impact on proliferation or apoptosis in normal human bronchial epithelial cells. The well-known biological role of IL-24 is to inhibit proliferation and promote apoptosis in a broad spectrum of cancer cells, and phase I clinical trial results suggested that a recombinant adenovirus overexpressing MDA-7/IL-24 (INGN-241) has shown certain clinical benefits for patients [35]. The function of IL-24 in cancer cells was inconsistent with our finding in normal human bronchial epithelial cells. Previously, several studies reported that IL-24 selectively induced growth arrest in cancer cells but had minimal effects on normal cells [22], and this evidence supported our data. As a pleiotropic cytokine, the physiological role of IL-24 seems to partly depend on its cellular source, target cells and the immune response of the host.

Prior studies have also shown that approximately 30–50% of myofibroblasts in murine lung tissues were derived from epithelial cells, which may undergo EMT in a murine asthma model [36, 37]. Our results demonstrated that IL-24 played a crucial role in EMT by upregulating the expression of vimentin and α-SMA and downregulating the level of E-cadherin in BEAS-2B cells via the STAT3 and ERK1/2 pathways. Moreover, we found that IL-24 was significantly elevated in the lung tissues surrounding the airway epithelium in an HDM-induced asthma murine model. Thus, we hypothesized that IL-24 could facilitate the phenotypic switch from epithelial cells to mesenchymal cells and induce EMT, thereby contributing to the development of airway remodeling in asthma. Indeed, in this study, targeted IL-24 downregulation by intranasal siRNA administration in asthmatic mice decreased in EMT marker expression and collagen deposition, which was accompanied by reduced TGF-β1 levels in BALF. Interestingly, intranasal administration of si-IL-24 and IL-37 failed to affect the serum level of TGF-β1. These data indicated that HDMs induced airway remodeling at least partially through IL-24. It is worth noting that IL-24 is expressed on not only the airway epithelium but also multiple immune cells [38–41]. The role and underlying mechanism of other IL-24-positive cells in asthma airway inflammation, EMT and airway remodeling remain to be elucidated. In fact, an association between IL-24 and tissue remodeling in many other diseases has been verified. For example, IL-24 is increased in the kidneys of newborn rats as well as at the edge of cutaneous rat wounds and played a role in the pathophysiology of congenital obstructive nephropathy (CON) by regulating tissue remodeling [42]. Another study showed that IL-24^−/−^ mice were protected against bleomycin-induced lung fibrosis by reducing TGF-β1 production and M2 macrophage infiltration [24]. A recent study reported that IL-24 treatment significantly increased the levels of TGF‑β and PDGF‑B in HT‑29 epithelial cells and upregulated the levels of ECM-associated genes in CCD‑18Co colon fibroblasts, indicating that IL-24 might contribute to the development of mucosal remodeling in IBD patients [19]. Contrary to the above results, Corbin et al. revealed that IL-24 inhibited EMT-related transcription factors and TGF-β in lung cancer cell lines and lung cancer models [43]. These contradictory results suggest that the regulatory effect of IL-24 on EMT may be related to the immune status of the disease. Collectively, these findings suggest that IL-24 or its receptors may be a promising therapeutic target for asthmatic airway remodeling.

In view of increasing evidence indicating that STAT3, ERK1/2 and other signaling pathways are related to EMT, we also verified multiple pathways in BEAS-2B cells that responded to IL-24 [44–46]. Our data showed that IL-24 selectively activated the JAK/STAT3 and ERK1/2 signaling pathways in BEAS-2B cells, which was consistent with other studies [20, 28]. Moreover, selectively antagonizing the ERK1/2 or STAT3 signaling pathway significantly alleviated the expression of IL-24-induced mesenchymal markers. In this study, we confirmed that IL-37 treatment downregulated vimentin and α-SMA expression in response to IL-24 stimulation by inhibiting the STAT3 and ERK1/2 signaling pathways in BEAS-2B cells. Furthermore, a similar therapeutic effect was verified in the animal model, as indicated by the alleviation of alleviating airway hyperresponsiveness and inflammatory cell infiltration, goblet cell hyperplasia, and subepithelial fibrosis and the reduction in TGF-β1 levels, and these results also hinted at the potential therapeutic effect of IL-37 on asthmatic airway remodeling.

Although our results provided new insight into the mechanisms of IL-24 in asthma airway remodeling, there are also some limitations and issues worthy of further in-depth research. First, in addition to bronchial epithelial cells, many other structural cells or immune cells involved in airway remodeling may express IL-24 receptors [38, 39, 41, 47], and the role and mechanism might not be entirely consistent. In addition, we cannot rule out the contribution of IL-24 secreted by other sources to amplifying the effect on airway inflammation, EMT and airway remodeling and is worthy of further investigation. IL-24 shares two heterodimeric receptors (IL-20R1 and IL-20R2) with IL-19 and IL-20 [48], and whether these receptors have similar or redundant biological effects on airway remodeling in airway epithelial cells or asthma models remains unclear and needs further examination. Although there are some limitations of our study, we provided evidence that deepens the understanding of the biological function linking IL-24 to the pathogenesis of asthma and might offer a novel perspective for the prevention and treatment of IL-24-associated EMT or airway remodeling in severe asthma. These topics will be deeply investigated by combining gene knockout or overexpression mouse models in our future study.

## Conclusions

In summary, we demonstrated that IL-24 contributed to airway remodeling by regulating the occurrence of EMT. Moreover, we further revealed that IL-37 protected against structural remodeling by inhibiting IL-24-mediated EMT by regulating the ERK1/2 and STAT3 signaling pathways, which might provide a theoretical basis for the development of IL-37 as a therapeutic agent and reveal possible drug targets to prevent and treat asthma airway remodeling.

## Supplementary Information

**Additional file 1: Fig. S1.** Interleukin-24 had no significant effect on proliferation, apoptosis, and cell cycle in BEAS-2B cells. (a) The effect of IL-24 on the cell viability of BEAS-2B cells was evaluated by CCK-8 assay followed by stimulation with different concentrations of IL-24 (0.1-100 ng/ml) for 24 h (n=5 wells / group). (b) The cells were stained with calcein AM and PI solution after treatment with 100 ng/ml IL-24 for 24 h. The green color indicates the live cells, and the red color represents the PI-positive nuclei. Scale bar=100 μm (×200). (c and d) After incubation with 10 or 100 ng/ml IL-24 for 24 h, flow cytometry was carried out to examine the cell apoptosis ratio. (e and f) The percentages of G0/G1, S and G2/M status were analyzed by flow cytometry after 10 or 100 ng/ml IL-24 treatment for 24 h. Bar diagrams and data are presented as the mean ± standard deviation (SD) from three replicate experiments. ns, no significant differences. **Fig. S2.** The effect of IL-24 and IL-37 on migration ability in BEAS-2B cells. (a) The effect of IL-24 on the migration of BEAS-2B cells was determined by wound healing assay. After stimulation with 100 ng/ml IL-24, scratches were captured at 0, 12 and 24 h. Scale bar=100 μm (×100). (b) Quantification of the wound healing assay showed the relative percentage of wound closure area. (c) The effect of IL-24 with or without IL-37 on the migration of BEAS-2B cells were evaluated by wound healing assay. After stimulation with 100 ng/ml IL-24 with or without 100 ng/ml IL-37, the scratches were captured at 0, 12 and 24 h. Scale bar=100 μm (×100). (d) Quantification of wound healing assay showed the percentage of wound closure area. Bar diagrams and data are presented as the mean ± standard deviation (SD) from three replicate experiments. * vs. control group; # vs. IL-24 group. *, #P < 0.05; **, ##P < 0.01; ***, ###P < 0.001.

## Data Availability

All data generated or analyzed during this study are included in this published article [and its supplementary information files].

## Abbreviations

IL: Interleukin
EMT: Epithelial–mesenchymal transition
HDM: House dust mite
FBS: Fetal bovine serum
DAPI: 4,6-Diamidino-2-phenylindole
ELISA: Enzyme-linked immunosorbent assay
JAK: Janus kinase
STAT: Signal transducer and activator of transcription
ERK1/2: Extracellular signal-regulated kinases
MAPK: Mitogen-activated protein kinase
NF-κb: Nuclear factor kB
DMSO: Dimethyl sulfoxide
α-SMA: Alpha smooth muscle actin
AHR: Airway hyper- responsiveness
BALF: Bronchoalveolar lavage fluid
Si-RNA: Small interfering RNA
