# Supplementary material for: IL-37 protects against airway remodeling by reversing bronchial epithelial–mesenchymal transition via IL-24 signaling pathway in chronic asthma
Source: Respir Res. 2022 Sep 13;23:244. doi: 10.1186/s12931-022-02167-7 (PMC9472332; doi:10.1186/s12931-022-02167-7)
Supplement: Supplementary file 1 — Additional file 1: Fig. S1. Interleukin-24 had no significant effect on proliferation, apoptosis, and cell cycle in BEAS-2B cells. (a) The effect of IL-24 on the cell viability of BEAS-2B cells was evaluated by CCK-8 assay followed by stimulation with different concentrations of IL-24 (0.1-100 ng/ml) for 24 h (n=5 wells / group). (b) The cells were stained with calcein AM and PI solution after treatment with 100 ng/ml IL-24 for 24 h. The green color indicates the live cells, and the red color represents the PI-positive nuclei. Scale bar=100 μm (×200). (c and d) After incubation with 10 or 100 ng/ml IL-24 for 24 h, flow cytometry was carried out to examine the cell apoptosis ratio. (e and f) The percentages of G0/G1, S and G2/M status were analyzed by flow cytometry after 10 or 100 ng/ml IL-24 treatment for 24 h. Bar diagrams and data are presented as the mean ± standard deviation (SD) from three replicate experiments. ns, no significant differences. Fig. S2. The effect of IL-24 and IL-37 on migration ability in BEAS-2B cells. (a) The effect of IL-24 on the migration of BEAS-2B cells was determined by wound healing assay. After stimulation with 100 ng/ml IL-24, scratches were captured at 0, 12 and 24 h. Scale bar=100 μm (×100). (b) Quantification of the wound healing assay showed the relative percentage of wound closure area. (c) The effect of IL-24 with or without IL-37 on the migration of BEAS-2B cells were evaluated by wound healing assay. After stimulation with 100 ng/ml IL-24 with or without 100 ng/ml IL-37, the scratches were captured at 0, 12 and 24 h. Scale bar=100 μm (×100). (d) Quantification of wound healing assay showed the percentage of wound closure area. Bar diagrams and data are presented as the mean ± standard deviation (SD) from three replicate experiments. * vs. control group; # vs. IL-24 group. *, #P < 0.05; **, ##P < 0.01; ***, ###P < 0.001. [file 12931_2022_2167_MOESM1_ESM.docx]

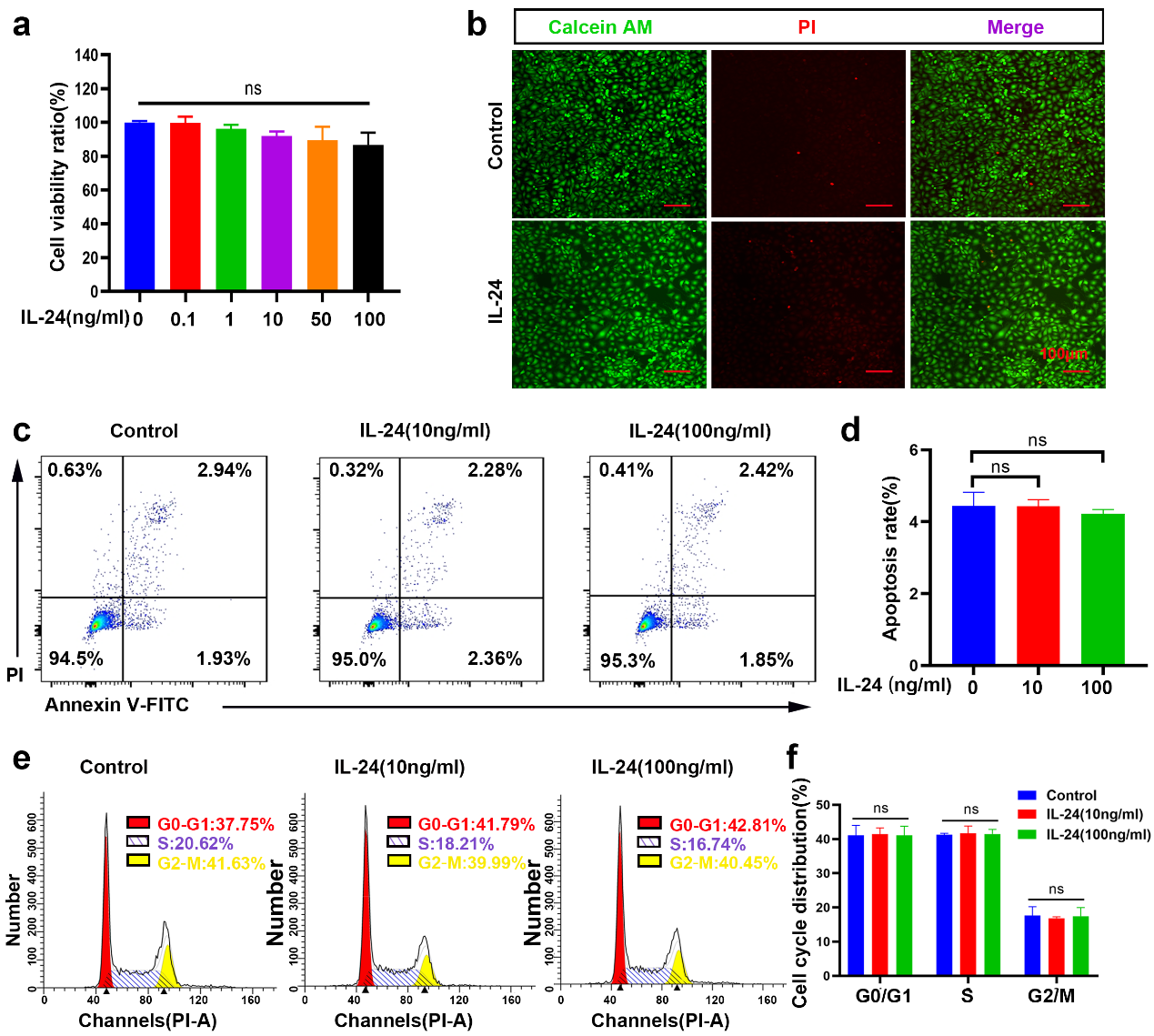


**Fig. S1.** Interleukin-24 had no significant effect on proliferation, apoptosis, and cell cycle in BEAS-2B cells. (**a**) The effect of IL-24 on the cell viability of BEAS-2B cells was evaluated by CCK-8 assay followed by stimulation with different concentrations of IL-24 (0.1-100 ng/ml) for 24 h (n=5 wells / group). (**b**) The cells were stained with calcein AM and PI solution after treatment with 100 ng/ml IL-24 for 24 h. The green color indicates the live cells, and the red color represents the PI-positive nuclei. Scale bar=100 μm (×200). (**c** and **d**) After incubation with 10 or 100 ng/ml IL-24 for 24 h, flow cytometry was carried out to examine the cell apoptosis ratio. (**e** and **f**) The percentages of G0/G1, S and G2/M status were analyzed by flow cytometry after 10 or 100 ng/ml IL-24 treatment for 24 h. Bar diagrams and data are presented as the mean ± standard deviation (SD) from three replicate experiments. ns, no significant differences.


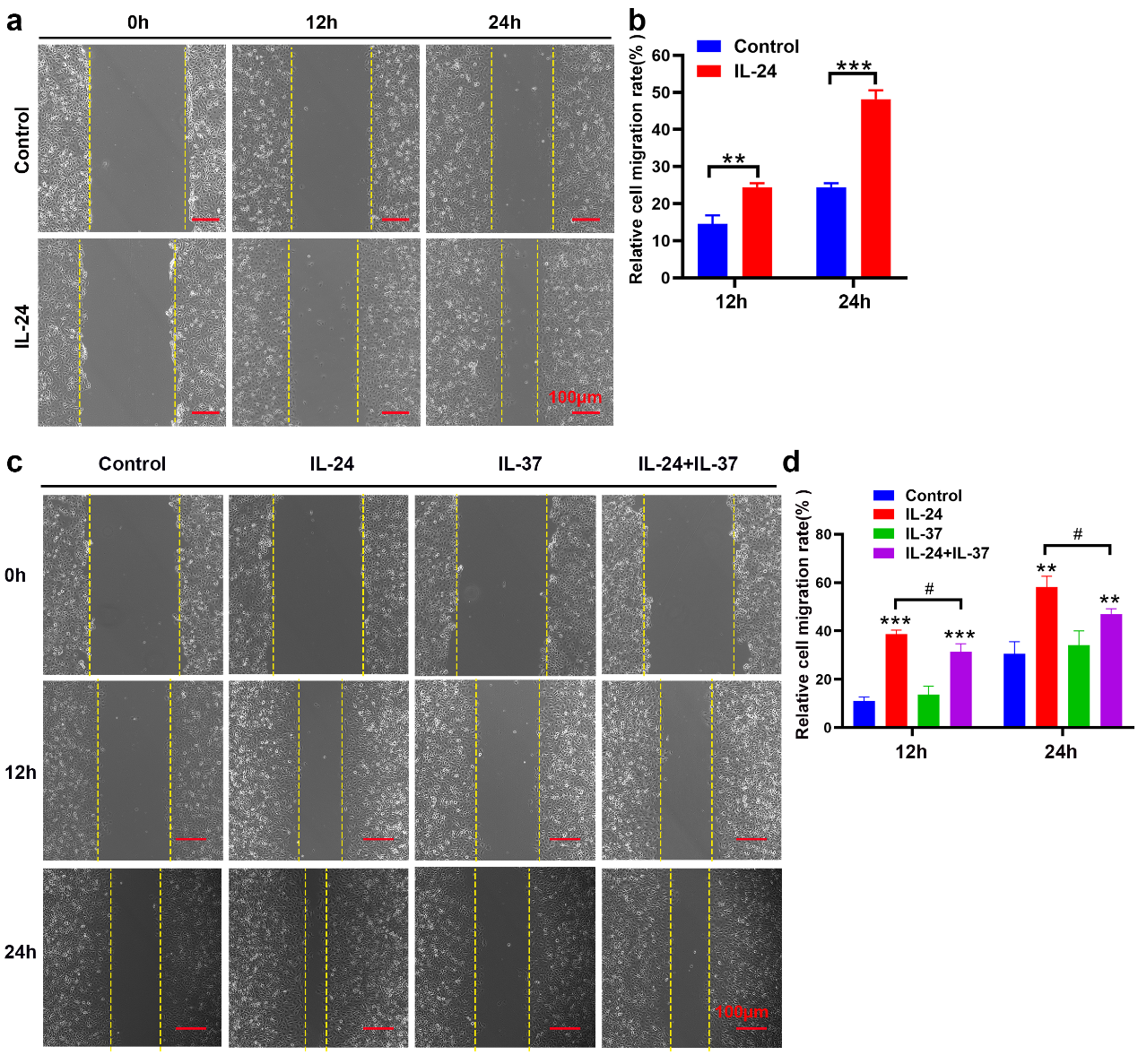


**Fig. S2.** The effect of IL-24 and IL-37 on migration ability in BEAS-2B cells. (**a**) The effect of IL-24 on the migration of BEAS-2B cells was determined by wound healing assay. After stimulation with 100 ng/ml IL-24, scratches were captured at 0, 12 and 24 h. Scale bar=100 μm (×100). (**b**) Quantification of the wound healing assay showed the relative percentage of wound closure area. (**c**) The effect of IL-24 with or without IL-37 on the migration of BEAS-2B cells were evaluated by wound healing assay. After stimulation with 100 ng/ml IL-24 with or without 100 ng/ml IL-37, the scratches were captured at 0, 12 and 24 h. Scale bar=100 μm (×100). (**d**) Quantification of wound healing assay showed the percentage of wound closure area. Bar diagrams and data are presented as the mean ± standard deviation (SD) from three replicate experiments. * vs. control group; # vs. IL-24 group. *^, #^*P* < 0.05; **^, ##^*P* < 0.01; ***^, ###^*P* < 0.001.
